# Evolutionary Pattern of Interferon Alpha Genes in Bovidae and Genetic Diversity of IFNAA in the Bovine Genome

**DOI:** 10.3389/fimmu.2020.580412

**Published:** 2020-09-30

**Authors:** Sunday O. Peters, Tanveer Hussain, Adeyemi S. Adenaike, Jordan Hazzard, Olanrewaju B. Morenikeji, Marcos De Donato, Sujay Paul, Masroor Babar, Abdulmojeed Yakubu, Ikhide G. Imumorin

**Affiliations:** ^1^Department of Animal Science, Berry College, Mount Berry, GA, United States; ^2^Department of Animal and Dairy Science, University of Georgia, Athens, GA, United States; ^3^Department of Molecular Biology, Virtual University of Pakistan, Lahore, Pakistan; ^4^Department of Animal Breeding and Genetics, Federal University of Agriculture, Abeokuta, Nigeria; ^5^Department of Biomedical Sciences, Rochester Institute of Technology, Rochester, NY, United States; ^6^Department of Biology, Hamilton College, Clinton, NY, United States; ^7^Tecnologico de Monterrey, Escuela de Ingenieria y Ciencias, Queretaro, Mexico; ^8^Department of Animal Science, Nasarawa State University, Lafia, Nigeria; ^9^School of Biological Sciences, Georgia Institute of Technology, Atlanta, GA, United States; ^10^Department of Biological Sciences, First Technical University, Ibadan, Nigeria

**Keywords:** interferons, type I, haplotypes, IFNAA, gene diversity

## Abstract

Interferons are secretory proteins induced in response to specific extracellular stimuli which stimulate intra- and intercellular networks for regulating innate and acquired immunity, resistance to viral infections, and normal and tumor cell survival and death. Type 1 interferons plays a major role in the CD8 T-cell response to viral infection. The genomic analysis carried out here for type I interferons within Bovidae family shows that cattle, bison, water buffalo, goat, and sheep (all Bovidae), have different number of genes of the different subtypes, with a large increase in the numbers, compared to human and mouse genomes. A phylogenetic analysis of the interferon alpha (IFNA) proteins in this group shows that the genes do not follow the evolutionary pattern of the species, but rather a cycle of duplications and deletions in the different species. In this study we also studied the genetic diversity of the bovine interferon alpha A (IFNAA), as an example of the IFNA genes in cattle, sequencing a fragment of the coding sequence in 18 breeds of cattle from Pakistan, Nigeria and USA. Similarity analysis allowed the allocation of sequences into 22 haplotypes. Bhagnari, Brangus, Sokoto Gudali, and White Fulani, had the highest number of haplotypes, while Angus, Hereford and Nari Master had the least. However, when analyzed by the average haplotype count, Angus, Bhagnari, Hereford, Holstein, Muturu showed the highest values, while Cholistani, Lohani, and Nari Master showed the lowest values. Haplotype 4 was found in the highest number of individuals (74), and in 15 breeds. Sequences for yak, bison, and water buffalo, were included within the bovine haplotypes. Medium Joining network showed that the sequences could be divided into 4 groups: one with highly similar haplotypes containing mostly Asian and African breeds, one with almost all of the *Bos taurus* American breeds, one mid-diverse group with mostly Asian and African sequences, and one group with highly divergent haplotypes with five N'Dama sequences and one from each of White Fulani, Dhanni, Tharparkar, and Bhagnari. The large genetic diversity found in IFNAA could be a very good indication of the genetic variation among the different genes of IFNA and could be an adaptation for these species in response to viral challenges they face.

## Introduction

Interferons (IFNs) are secreted signaling proteins made and released by host cells in response to the presence of pathogens such as viruses, bacteria, parasites, or tumor cells (1). Interferons allow for communication between cells to trigger the protective defenses of the immune system that eradicate pathogens or tumors. Interferons are named after their ability to “interfere” with viral replication within host cells (2). IFNs have other specific functions which includes activation of immune cells, such as natural killer cells and macrophages (3); increase in recognition of infection or tumor cells by up-regulating antigen presentation to T lymphocytes; and increase in the ability of uninfected host cells to resist new infection by virus. Some of the symptoms associated with IFNs production during infection are aching muscles and fever (2).

Three types of IFNs have been described, Type I, II, and III or IFN-like cytokines. Type I IFNs is the most diverse family with several closely related subtypes, with 8 subclasses been described in different mammalian species: IFNA (alpha), IFNB (beta), IFND (delta), IFNE (epsilon), IFNK (kappa), IFNT (tau), IFNW (omega), and INFZ (zeta or limitin), which all being recognized by the conjunction of IFNAR1 and IFNAR2 (4). Type II IFN consists of IFNG (gamma) only (5). IFNA and IFNB represent the major interferons synthesized by leukocytes and fibroblasts, respectively, after challenge with viruses, double-stranded RNA, or other inducers (6). The distribution of the subtypes of type I IFNs among the eutherians is different depending on the taxa, with IFNA, IFNB, IFNE, IFNK, and IFNW being the only types found in humans, which suggests that the diversification of the family seems to have arisen independently in each species (4, 7). There are multiple IFNA genes reported in humans and many other species (8). Type I IFN genes in *Bos taurus* has undergone significant rearrangement and expansion compared to human and mouse (3, 9, 10).

As a major component of the innate immune system protecting against viral infection, the expression of Type I IFNs is induced by viral challenges, and the Toll-like receptors play an important role in the expression of IFNs (11). The IFNA family is released by almost all cell types and a few of the human family members, specifically human IFNA2a and IFNA2b, are currently approved for treatment of a range of viral diseases including hepatitis B and C, condylomata acuminate (genital warts), and AIDS-related Kaposi sarcoma (12). Recombinant bovine IFNA proteins (IFNAE) showed to inhibit the cytopathic effect of the vesicular stomatitis virus against Madin-Darby bovine kidney (MDBK) cells (13). Even though IFNAs and IFNBs were successfully purified since early 1980s (12), there are many gaps of information in terms of their function. Much of the knowledge about type I IFN effects on the replication and pathogenesis of virus infection for *in vivo* models comes from deletion of IFNAR1, which lack IFN signaling (14), which do not allow for the study of the different type I IFN subtypes.

IFNA subtypes limited Chikungunya virus replication and spread, whereas IFNB functioned primarily to limit inflammation by modulating neutrophil accumulation at the site of infection (14) It is known that IFNA, by the binding to IFNAR1, can initiate the signaling cascade that activates STAT1 and STAT2, which then form the transcription factor complex ISGF3, which includes both, as well as IRF9, which increasing the expression of IFN-stimulated genes (ISGs) and turns triggers the immune response; However, when there is a prolonged stimulation of IFNA, ISGs can be induced by a STAT2-dependent, STAT1-independent pathway (15).

Although annotation of interferon genes has been documented in various species of animals, little is known about the variations, and evolutionary pattern exhibited by Type I IFNs genes in Bovidae, in general, and more specifically, in cattle, especially in view of the publication of the new *de novo* bovine genome assembly (ARS-UCD1.2), which includes 244 Gb of new PacBio sequence with an average insert size of 20 kb and 340 Gb of new TruSeq PCR-free Illumina sequence with an average insert size of 550 bp, as well as half a million new reads from 23 tissues from RNA-seq PacBio sequencing (16). Additionally, even though high genetic variation is an important factor that favors a greater range of pathogens to be recognized, especially in African and Asian breeds, which are more challenged by disease pathogens compared to other continental populations, there is not a single study reporting the haplotype variation in cattle about any of interferon genes, and there are only few sequences reported in GenBank of the IFNA genes. Therefore, the study of the evolutionary pattern of IFNA genes in the Bovidae, which is most diversified interferon subtype in all the mammalian species, and the genetic variation of a representative of this subfamily, such as IFNAA, in breeds from different regions, could help to understand how genetic variation pattern can be affected by the environmental differences, the selection objectives of each breed and their exposure to pathogens, especially virus.

## Materials and Methods

### Evolutionary Pattern of Type I IFN Proteins in Bovidae

The analysis of the type I IFNA proteins were studied in the genomes of the members of Bovidae that have genome sequence assemblies available, such as cattle: *Bos taurus* (ARS-UCD1.2), bison: *Bison bison bison* (Bison_UMD1.0), water buffalo: *Bubalus bubalis* (ASM312139v1), sheep: *Ovis aries* (Oar_rambouillet_v1.0) and goat: *Capra hircus* (ASM170441v1), as well as those of white-tailed deer: *Odocoileus virginianus texanus* (Ovir.te_1.0), and pig: *Sus scrofa* (Sscrofa11.1), as outgroups.

Phylogenetic analysis of the bovine type I IFNs proteins was carried out using the Maximum Likelihood method based on the General Time Reversible model with a discrete Gamma distribution to model evolutionary rate differences among sites. Since most genes have temporary LOC names, we recoded them according to their phylogenetic relationship, but keeping the ones that have previously assigned names (Supplementary Table 1). The phylogenetic analysis of the Bovidae subtype IFNA proteins were analyzed likewise.

### Sequencing the Bovine IFNAA Gene

Blood samples were collected from 18 breeds of cattle from Nigeria (Africa), Pakistan (Asia), and the United States (Table 1), according to the protocol approved by Institutional Animal Use and Care Committee of Cornell University. The selection of animals and collection of samples was carried out as described in a previous study (17). Genomic DNA was purified using the organic extraction method described by Babar et al. (18).

**Table 1:** Characterization of the DNA polymorphisms found in the bovine IFNAA gene in the cattle breeds.

**Breed**	***N***	**S**	**Hap**	**Hd**	**Pi**	**k**	**NSS**	**SS**	**Tajima D**
Achai	13	17	6	0.895	0.0084	1.31	214.61	73.39	−0.266
Angus	3	15	2	1.000	0.0343	5.00	230.17	75.83	n.a.
Bhagnari	15	25	9	0.971	0.0358	1.67	196.89	70.11	−2.384^*^
Brangus	14	26	8	1.000	0.0287	1.86	226.98	67.02	0.415
Cholistani	17	12	5	0.426	0.0034	0.71	218.90	81.10	−1.976^*^
Dajal	17	23	7	0.669	0.0196	1.35	206.30	75.70	−1.168
Dhanni	15	24	7	1.000	0.0208	1.60	211.70	67.30	−0.134
Hereford	3	16	2	1.000	0.0355	5.33	228.33	74.67	n.a.
Holstein	6	19	4	1.000	0.0239	3.17	225.31	71.69	0.288
Lohani	16	18	6	0.767	0.0146	1.13	212.95	81.05	0.244
Muturu	8	11	5	0.795	0.0062	0.98	158.19	45.81	−0.700
N'Dama	15	45	6	0.893	0.0452	3.00	188.69	63.31	−1.291
Nari Master	8	7	3	0.975	0.0056	0.88	222.17	77.83	−0.503
Red Sindhi	13	22	7	0.956	0.0152	1.69	211.88	76.12	−0.384
Sahiwal	8	10	4	0.767	0.0041	0.95	224.44	72.56	−1.422
Sokoto Gudali	15	17	8	0.752	0.0108	1.13	155.22	45.78	−0.174
Tharparkar	13	25	7	0.975	0.0181	1.92	195.41	71.59	−0.569
White Fulani	14	35	8	0.917	0.0208	2.50	158.12	54.88	−1.525
Overall	213	77	22	0.606	0.0190	0.59	58.89	16.11	−1.294

*N, number of sequences; S, number of polymorphic sites; Hap, number of haplotypes; Hd, haplotype (gene) diversity; Pi, nucleotide diversity, k, average number of nucleotide differences; SS, synonymous substitutions; NSS, non-synonymous substitutions; n.a, not available*.

* *Statistically significant values*.

DNA quantity, quality and integrity were checked using NanoDrop2000 (Thermo Scientific, Wilmington, DE) and gel electrophoresis. DNA concentration were adjusted to 50 ng/μL.

Specific primers (IFNAA-F: AAAGCATCTGCAAGGTCCCCGAT, IFNAA-R: TCCTCCTGCGTCAGACAGGCTT) were designed using the Primer3 software (19) with the mRNA sequence from GenBank (NM_001017411.1), in order to amplify a partial CDS fragment of 401 bp of the *Bos taurus* IFNAA gene, which covered 66.7% of the coding sequence.

The amplification was carried out using the Applied Biosystem GeneAmp9700 system with a total volume of 25 μL, using 50 ng of gDNA, 0.1 pM of each primer, 10 μM of dNTPs, 2.5 mM of MgCl_2_ and 1.5 U of Taq DNA polymerase (Fermentas, Thermo Fisher Scientific Inc. USA). The PCR conditions was carried out with an initial denaturation at 94°C for 5 min, 35 cycles of denaturation at 94°C for 30 s, annealing at 60°C for 30 s, and extension at 72°C for 30 s followed by final extension at 72°C for 7 min. The fragment sequencing was carried out in the Cornell University Core Lab using a Genetic Analyzer 3130xL (Applied Biosystems, Inc., Foster City, CA).

After the analysis of the sequences, only 313 base pairs of the IFNAA gene were included. The sequence alignment was carried out using MUSCLE software (20) with adjustment by visual revision. In addition to the sequences obtained in this study, we include in the analyses all the sequences found in GenBank for the IFNAA gene in the species of the Bovidae family: *Capra hircus* (NM_001285704.1, XM_005683607.2, XM_018044725.1, XM_018045635.1, XM_018052805.1, XM_018052806.1, XM_018052808.1, and XM_018052815.1), *Ovis aries* (AY802984.1, AY802985.1, AY802986.1, XM_004004404.3, XM_004005319.3, XM_012108174.1, XM_012126940.2, XM_012126974.1, XM_012126994.2, XM_012172382.2, XM_012172384.2, XM_012172388.2, XM_012173131.2, and XM_015092790.1), *Bubalus bubalis* (XM_025279284.1), *Bison bison* (XM_010845557.1), *Bos mutus* (XM_005903775.1) and including the few cattle sequences: *B. taurus* (NM_001017411.1, M10952.1, EU276064.1, and Z46508.1), *B. indicus* (XM_010845557.1), and *B. taurus* x *B. indicus* (XM_027535645.1).

### Population Genetic Analysis Using the Bovine IFNAA Sequence

**Figure 1:**
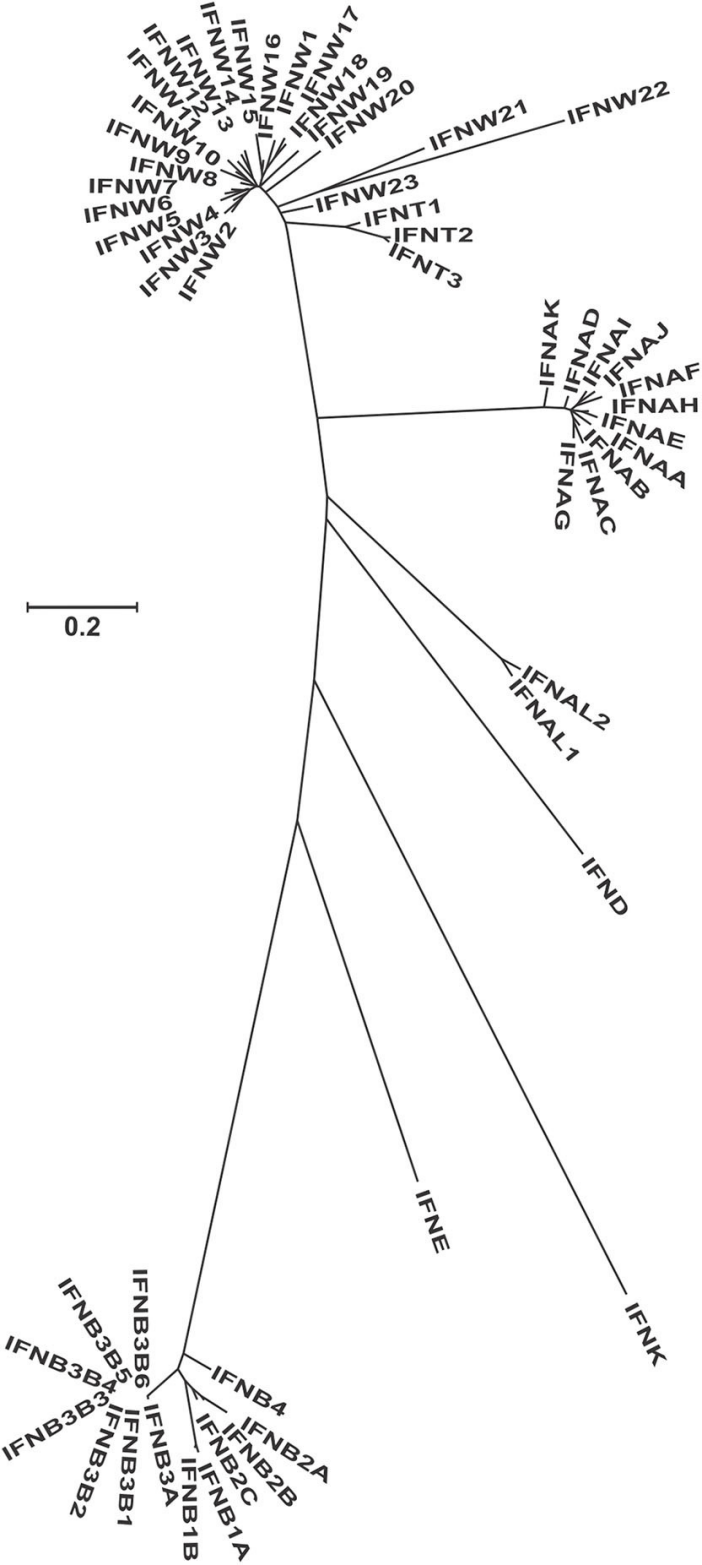
Molecular phylogenetic analysis of all the protein sequences from the GenBank for the type I interferons in the bovine genome. This unrooted tree was constructed using the Maximum Likelihood method based on the General Time Reversible model. The tree with the highest log likelihood (−5538.07) is shown. A discrete Gamma distribution was used to model evolutionary rate differences among sites [5 categories (+G, parameter = 2.16)]. The tree is drawn to scale, with branch lengths measured in the number of substitutions per site. Since most genes have temporary LOC names, we recoded them according to their phylogenetic relationship, but keeping the ones that have assigned names (Supplementary Table 1).

Sequence variation and haplotype structure were calculated using DnaSP version 5.10.01 (21) in order to analyze the genetic diversity within and between breeds for this gene. This analysis allowed us to calculate the rate of synonymous and non-synonymous substitutions (dN/dS), number of polymorphic sites, haplotypes and nucleotide and haplotype diversity. Tajima's *D*-test (22) was used to test the neutrality of the polymorphic sites. Here, a haplotype was defined as a group of sequences not differing more than 0.02 substitutions per site and/or showing a monophyletic pattern (Figure 1), as previously reported (17).

Phylogenetic networks of the haplotypes were analyzed by the median-joining network method using Network software (23), version 5.1.0.0 (http://www.fluxus-engineering.com/sharenet.htm). The Maximum Likelihood method, based on the General Time Reversible model (24), was used to construct a phylogenetic tree by MEGA software version X (25), using 1000 iterations to calculate bootstrap value (26) as the statistical support of the branches in the tree.

ARLEQUIN software, version 3.001 (27), was used to calculate the molecular diversity indices, and the population pairwise F_ST_ values, after 1,000 iterations. Analysis of molecular variance (AMOVA) was used to test significant differences in the IFNAA gene diversity between cattle breeds. R program (28) was used to generate F_ST_ plots and to carry out the principal component analysis through ade4 package. Marker counts based on polymorphic sites of the sequences were extracted before performing the analysis.

Amino acid sequences for IFNAA were inferred using the translation function of the MEGA software version X. The average number of non-synonymous (d_N_) and synonymous (d_S_) substitutions per site and the standard errors were calculated using the modified Nei and Gojobori (29) model for each continental cattle and combined breeds altogether. The Jukes-Cantor correction was used to correct for multiple substitutions at the same site. The ratio of d_N_ and d_S_ substitutions was tested for departure from neutral expectations using *Z*-statistic in MEGA version X.

The functional effects of the nsSNPs of the bovine IFNAA gene were predicted computationally using PROVEAN and Polyphen-2. PROVEAN and Polyphen-2 models were applied as described in an earlier study (30).

## Results

The BLAST search of the interferon proteins in the species of *Bos taurus, Bison bison, Bubalus bubalis, Capra hircus, Ovis aries*, shows a large expansion of type I IFNs genes, compared to human and mouse (Table 2). The main expansions are seen in subtypes alpha-like, beta, tau, and omega. In the white-tailed deer, the expansion of the subtypes alpha-like, beta and omega was compensated by a reduction in the alpha subtype, while in pigs, the expansion was limited to alpha, delta and omega. Only epsilon and kappa were found as a single gene in all the species. In all these species, the coding sequence was found in a single exon.

**Table 2:** Number of functional type I interferon genes annotated in the species of Bovidae and other mammalian species for comparison.

**Species**	**Common name**	**IFNA**	**IFNAL**	**IFNB**	**IFND**	**IFNE**	**IFNK**	**IFNT**	**IFNW**	**Total**
*Bos taurus*	Bovine	11	2	13	1	1	1	3	23	55
*Bison bison*	American Bison	10	1	5	0	1	1	6	21	45
*Bubalus bubalis*	Water buffalo	14	1	6	0	1	1	2	17	42
*Capra hircus*	Goat	9	0	8	1	1	1	2	31	53
*Ovis aries*	Sheep	7	6	11	1	1	1	9	26	62
*Odocoileus virginianus*	White tailed deer	3	3	6	0	1	1	0	5	19
*Sus scrofa*	Pig	16	1	1	12	1	1	0	7	39
*Homo sapiens*(4)	Human	13	0	1	0	1	1	0	1	17
*Mus musculus*(9)	Mouse	11	0	1	0	1	1	0	1	15

Because the bovine genome is the best assembled genome after the human and mouse, we conducted a more detailed study of the type I IFN genes. The phylogenetic relationship among the different proteins show very clear clusters for alpha, omega, alpha-like, delta, kappa, epsilon, and beta (Figure 1, Supplementary Table 1). Tau is the only subtype that seems like a more diversified form of an omega gene than a different subtype. In the beta subtypes, identical copies of IFNB3B (named 1–6) have been found, evidence of a very recent duplication event, since no differentiation has occurred among the copies. IFNK is the most differentiated type I gene and is the only gene located in a different location in the genome, while the rest of type I IFNs are located in a single region. This is also seen in all other species where the genes have been assigned to specific chromosomal regions.

**Figure 2:**
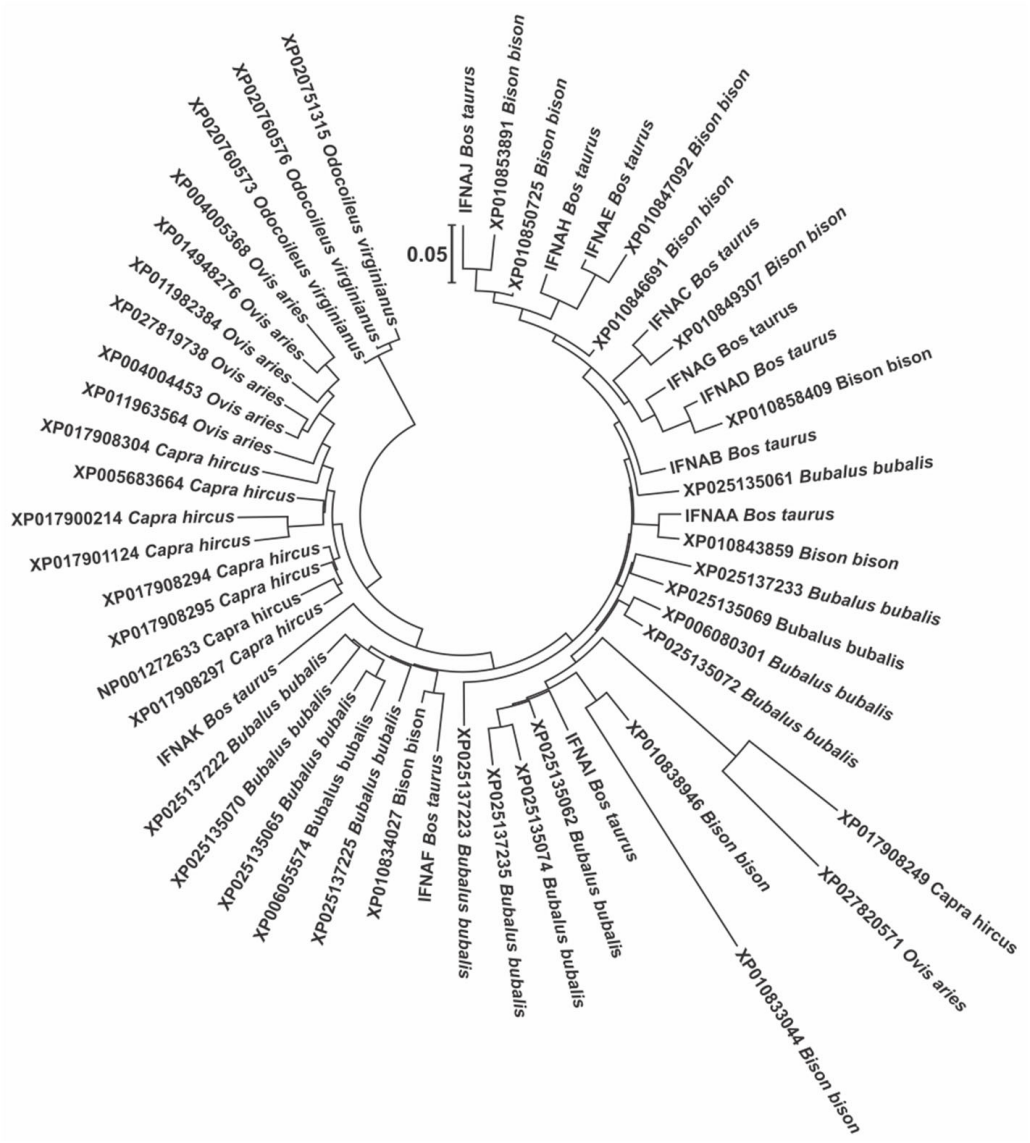
Molecular phylogenetic analysis of all the protein sequences from the GenBank for the IFNA genes in the members of the Bovidae, using the genes of the white-tailed deer as outgroups to root the tree. This tree was constructed using the Maximum Likelihood method based on the General Time Reversible model. The tree with the highest log likelihood (−3004.05) is shown. A discrete Gamma distribution was used to model evolutionary rate differences among sites [5 categories (+G, parameter = 0.86)]. The tree is drawn to scale, with branch lengths measured in the number of substitutions per site.

Being IFNAs the only subtype present in multiple copies in most species, it is very interesting to study them in terms of its evolution, function and variation within each species. In this sense, the phylogenetic analysis of the IFNAs in the Bovidae show a very large variation among the species, with no correlation with the known phylogenetic relationship among the Bovidae species (Figure 2). In fact, the genes are separated into two clusters, one that contains all the genes of bovine, bison, and buffalo (Bovinae subfamily), except for one gene of goat and one of sheep, and another cluster with the rest of the genes of goat and sheep (Caprinae subfamily). There is a clear indication that lower taxa (species, genus, subfamily) seem to have multiple events of gene deletion and duplication.

To study the variation of the IFNAs in the bovine genome, we chose to study in more detail the IFNAA gene to ascertain the level of diversity that existed among the cattle breeds from three continents. Thus, we analyse the sequence of a 313 bp fragment of this gene, representing 54% of the coding sequence, containing the complete region binding to IFNAR-1 and most of the region binding to IFNAR-2. The analysis of the variability of the whole coding sequence, using the only 6 *B. taurus* and *B. indicus* sequences, as well as the IFNAA published sequences for *B. mutus, B. bison, B. bubalis, C. hircus*, and *O. aries*, shows that the sequence analyzed here contains 60.7% of the variable sites of the whole coding sequence (data not shown). For this, we believe that this fragment is representative to study the evolutionary pattern for the entire gene.

**Figure 3:**
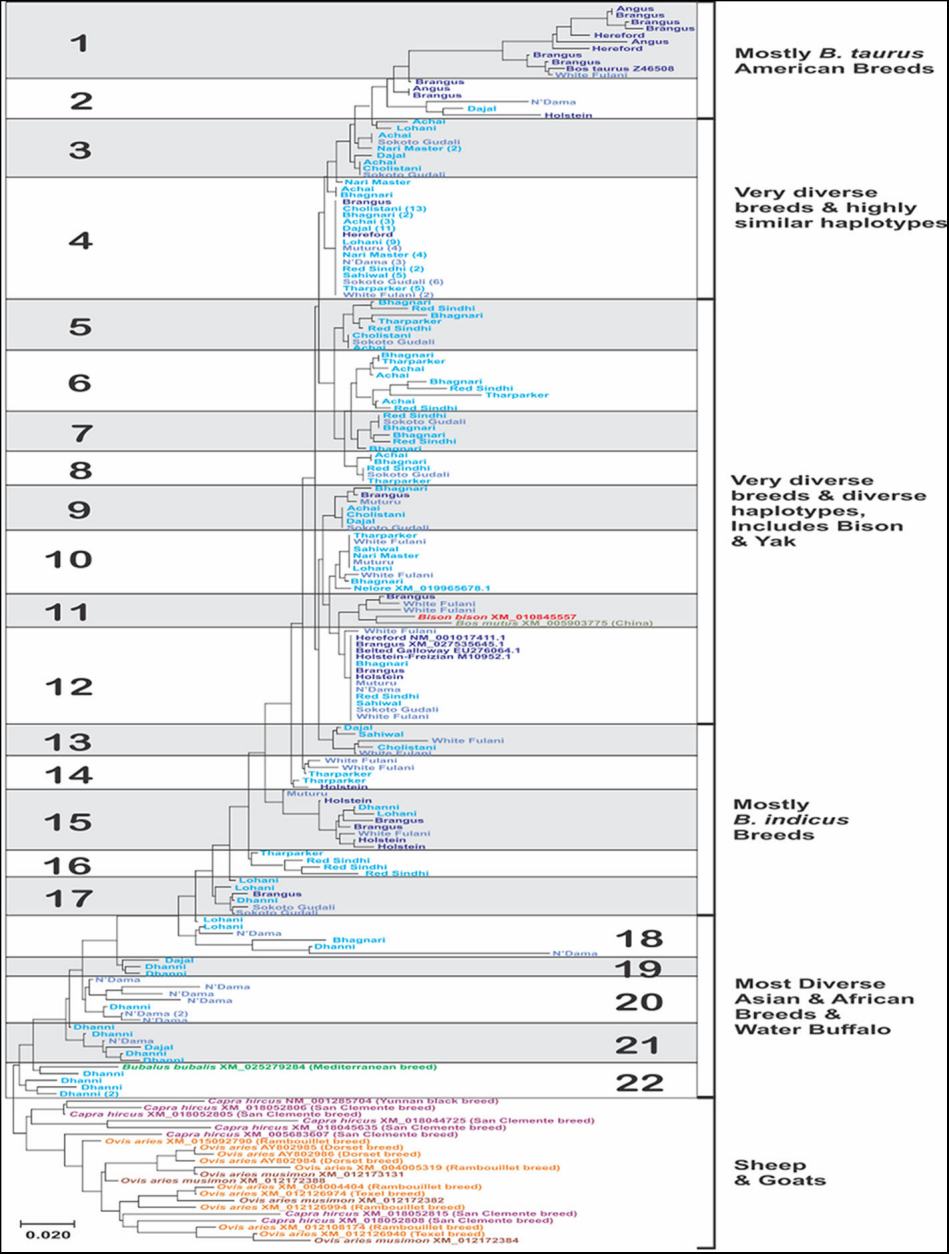
Molecular phylogenetic analysis of the sequences of a fragment of 313 bp from the coding region of the bovine IFNAA in 213 individuals from 18 breeds of cattle, constructed inferred using the Maximum Likelihood method based on the General Time Reversible model. The tree with the highest log likelihood (−2265.83) is shown. A discrete Gamma distribution was used to model evolutionary rate differences among sites [5 categories (+G, parameter = 0.17)]. The tree is drawn to scale, with branch lengths measured in the number of substitutions per site. The numbers in parenthesis in some of the sequences represent the number of individual sequences that had 100% identity.

A total of 22 different haplotypes have been proposed (Figure 3), according to their nucleotide identity values of the sequences for the IFNAA gene. The bootstrap values were highly variable and most of them below 50%, which is expected because of the high variability of the sequences. The sequences generated in this study have been submitted to GenBank (MH478673–MH478911).

The breeds with the highest number of haplotypes (Supplementary Tables 1, 2) were Bhagnari (9), Brangus, Sokoto Gudali and White Fulani (8 each), while the least numbers were found in Angus (2), Hereford and Nari Master (3 each). However, this was influenced by the number of individuals analyzed per breed, thus when we analyze the average haplotype count (AHC), we found that the breeds with the highest values were instead Angus, Bhagnari, Hereford, Holstein, Muturu (0.600–0.667), while Cholistani, Lohani, and Nari Master showed the lowest values (0.294–0.375). Additionally, the highest values for haplotype diversity was found in Angus, Hereford, Holstein, Dhanni and Brangus (1.000), while Cholistani had the lowest (0.426). Haplotype 4 was found in the highest number of individuals (74), and in 15 breeds. Haplotype 22 was found in a single breed. The second most common haplotype was 12, present in 9 breeds. Only the haplotype 22 was found in a single breed.

Interestingly, the sequences of the species of wild yak, bison and water buffalo, belonging to the Bovinae, were included within the bovine haplotypes (Figure 3), with bison and yak showing haplotype 11, also found in Brangus and White Fulani, while water buffalo showed haplotype 22, which was found in only Dhanni. The sequences found for goat and sheep showed high divergence among them, with only three clusters of sheep sequences showing higher identity values. Goat sequences were more divergent than sheep, even though they belonged to only two breeds.

Of the 77 total variable sites observed in the sequences, considering all breeds, 49 were polymorphisms found in at least 1% of the individuals (more than 3 individuals) and at least in two breeds. The highest numbers of variable sites (S, Table 2), an indication of within-breed variation, were found in N'Dama and White Fulani (45 and 35, respectively), and the average number of nucleotide differences (k), was higher in White Fulani, N'Dama, Holstein, Angus and Hereford (2.50-5.33). On the other hand, Nari Master, Sahiwal, Muturu, and Cholistani showed the lowest values of S (7–12). These results agree with the phylogenetic relationships among haplotypes (Figure 3).

The number of non-synonymous substitutions (dN) for all of the breeds were higher than the synonymous substitutions (dS) with Angus having the highest value of dN (230.17) and Sokoto Gudali had the least value (155.22) while Cholistani and Lohani had the highest values of dS (81.10 and 81.05, respectively) and Muturu and Sokoto Gudali showing the lowest values (45.81 and 45.78, respectively).

**Figure 4:**
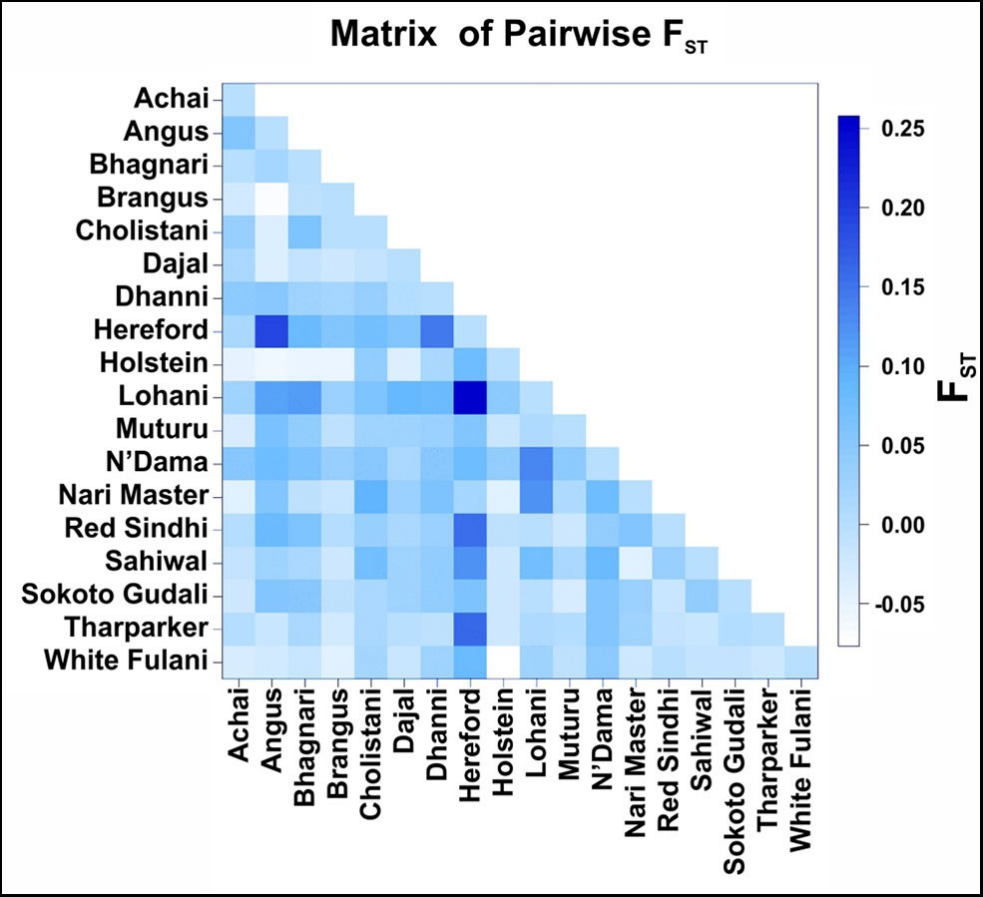
Pairwise F_ST_ comparison between cattle breeds. The F_ST_ values are coded with a color code shown in the legend on the right side.

The highest level of variation found in the AMOVA analysis (85.4%) was within breeds (Table 3). Very low values of pairwise F_ST_ (Figure 4) was found between the American breeds Angus and Brangus (−0.068), as well as between the Asian breeds Sahiwal and Tharparkar (−0.015) and the African breeds Muturu and Sokoto Gudali (0.030) and N'Dama and Sokoto Gudali (−0.035). All of the negative values are not different from 0.

**Table 3:** AMOVA analysis of the variation found within and among cattle breeds studied.

**Source of variation**	**Degree of freedom**	**Variance components**	**Percent of variation**
Among population	2	8.5819	14.63
Within population	238	50.0975	85.37

When comparing breeds from different regions, Pairwise F_ST_ ranged from −0.077 (between Holstein and White Fulani) to 0.258 (between Hereford and Lohani). In this study, the fixation index F_ST_ was estimated to be 0.146.

**Figure 5:**
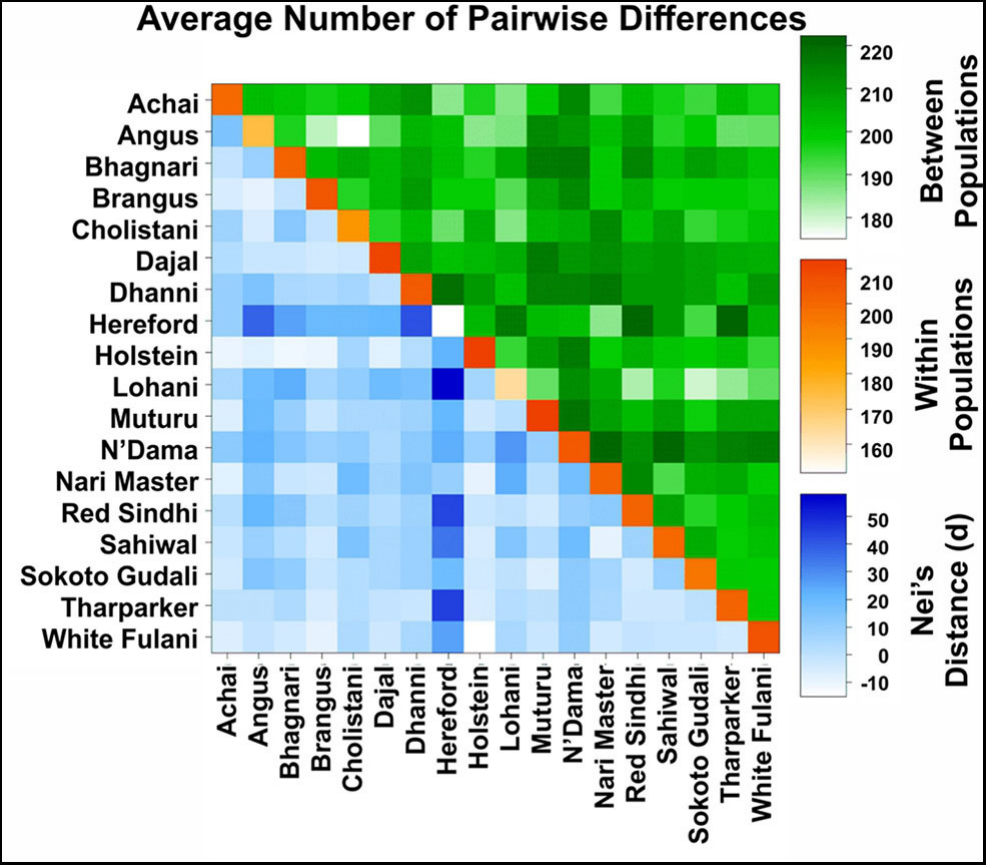
Average number of pairwise differences between cattle breeds. The average number of pairwise differences between each cattle breed in the upper half of the matrix (green). The average number of pairwise differences within each cattle breed is shown in the diagonal (orange). The lower half of the matrix (blue) showed the corrected average number of pairwise differences between cattle breeds.

The Average number of pairwise differences (K) within breeds varied from 151.67 in Hereford to 212.68 in Muturu (Figure 5, diagonal squares). In American breeds, K values between breeds (Figure 5, squares above the diagonal) ranged from 181.49 (between Angus and Brangus) to 204.67 (between Hereford and Holstein). K values in Asian breeds ranged from 182.93 (between Lohani and Red Sindhi) to 220.09 (between Nari Master and Sahiwal). In African breeds, Muturu and N'Dama (208.99) showed the highest value of K, while Muturu and Sokoto Gudali showed the lowest value (197.24).

**Figure 6:**
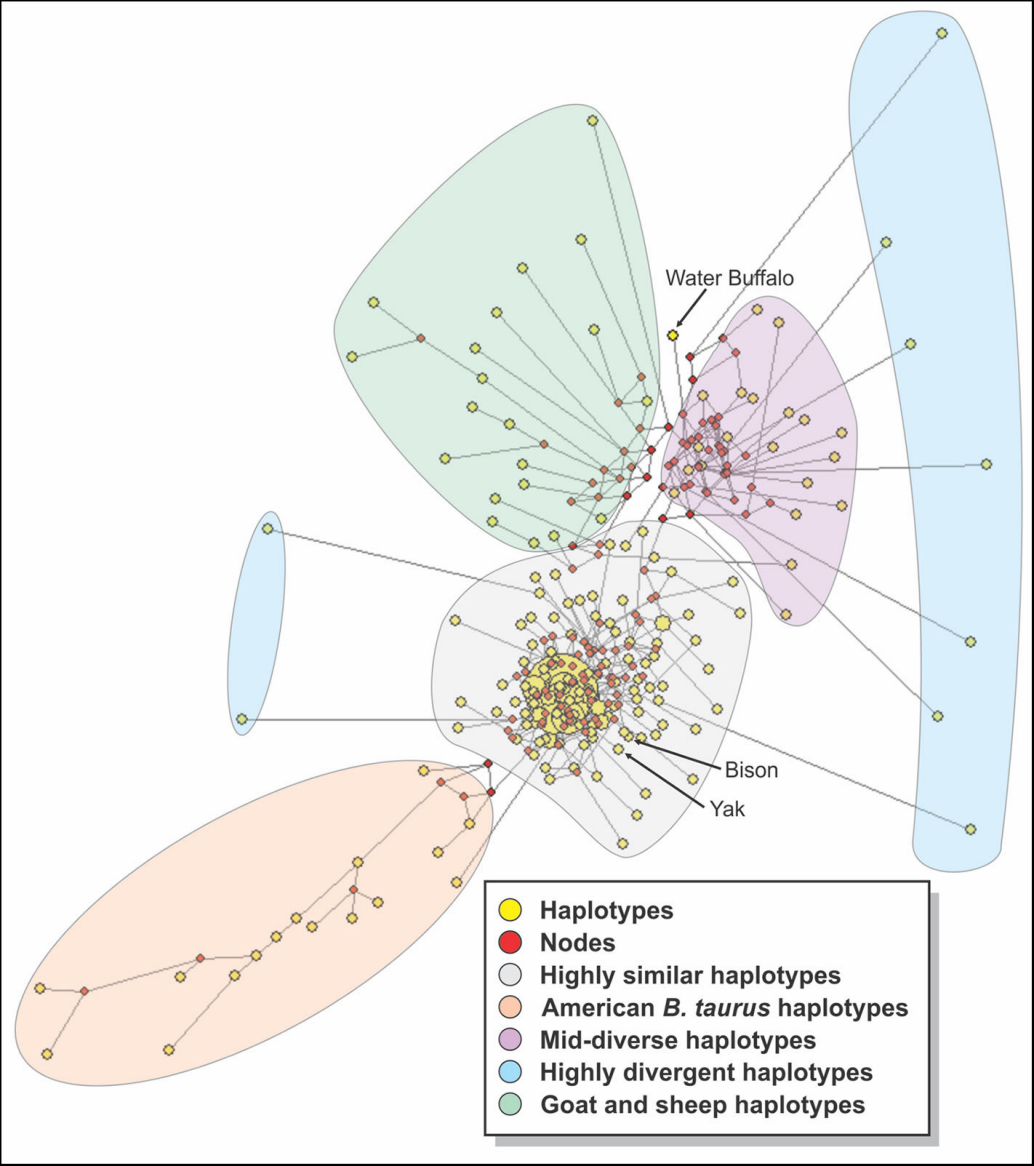
A median joining network constructed with all the sequences of cattle studied. Yellow circles represent haplotypes and have a size proportional to their frequency. The red squares represent the median vector; branch length (lines) is proportional to the number of mutations.

Medium Joining network obtained from haplotypic data (Figure 6) showed that the sequences could be divided into 4 groups: a group with highly similar haplotypes containing mostly Asian and African breeds from which the rest of the haplotypes seem to be derived, a group with almost all of the American breeds, a mid-diverse group with mostly Asian and African sequences, a group of highly divergent haplotypes with five N'Dama sequences and one of each of White Fulani, Dhanni, Tharparkar, and Bhagnari breeds. The sheep and goat sequences formed their own group, while water buffalo diverged from the mid-diverse group. The sequences for bison and yak were grouped in the highly similar group.

**Figure 7:**
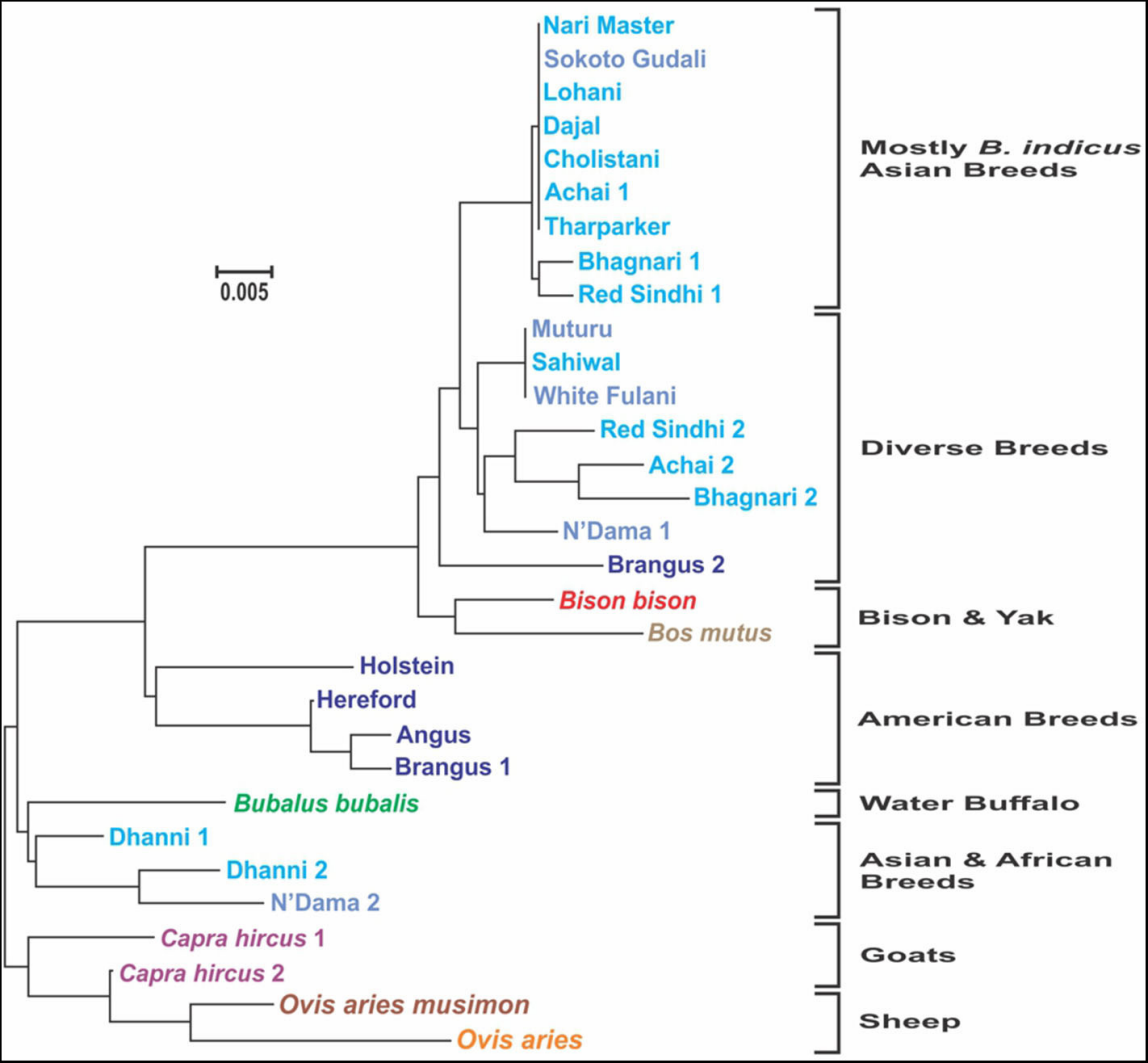
Molecular phylogenetic analysis of the consensus sequences for each breed, constructed inferred using the Maximum Likelihood method based on the General Time Reversible model (24), carried out in MEGA [Version 10.1.7, Kumar et al., (25)]. The tree with the highest log likelihood (−903.7286) is shown. A discrete Gamma distribution was used to model evolutionary rate differences among sites [5 categories (+G, parameter = 0.1688)]. The tree is drawn to scale, with branch lengths measured in the number of substitutions per site. In the breeds showing sequences too divergent, two consensus sequences were generated.

**Figure 8:**
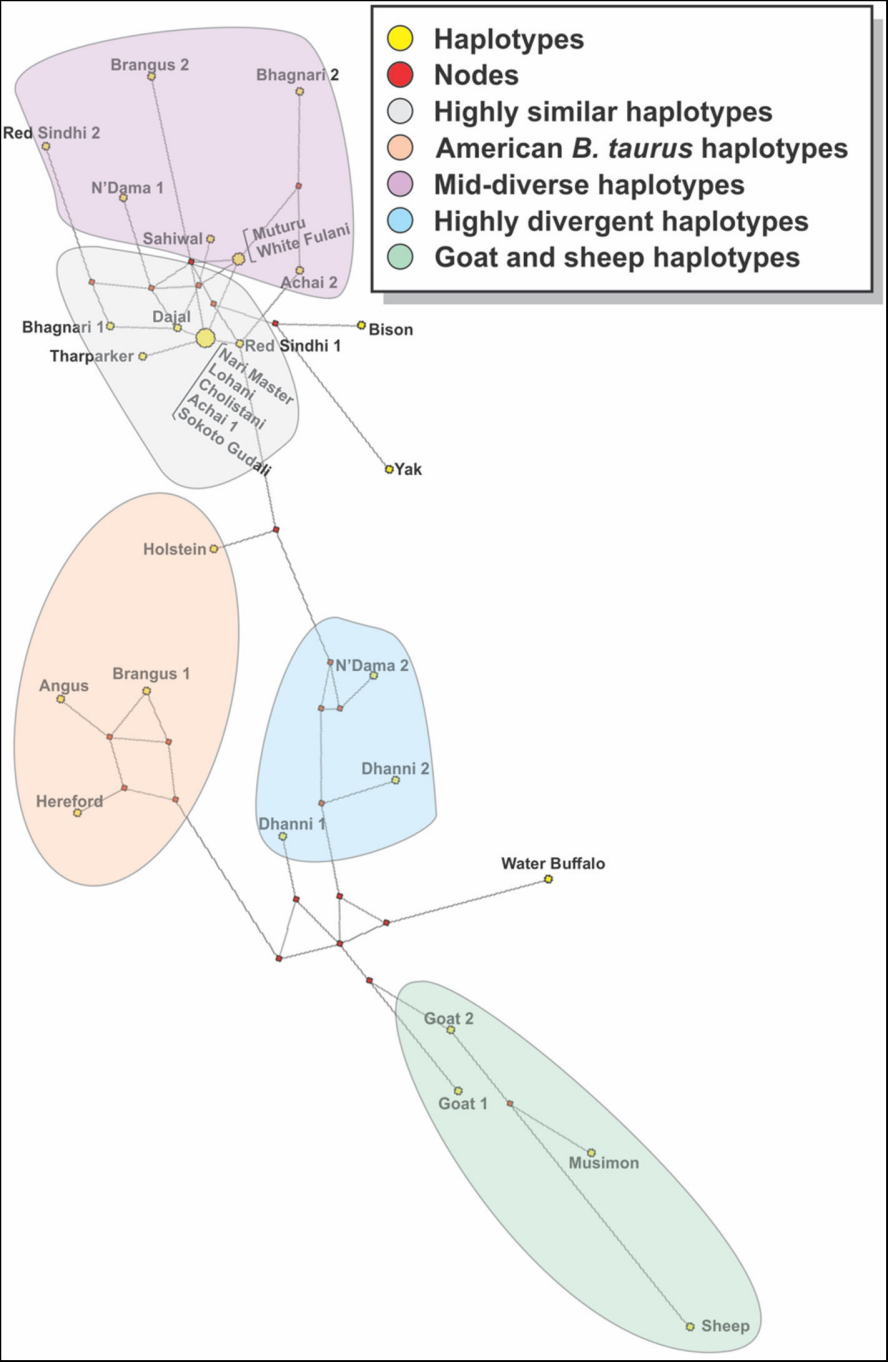
A median joining network constructed with the consensus sequences of cattle breed studied. Yellow circles represent haplotypes and have a size proportional to their frequency. The red squares represent the median vector; branch length (lines) is proportional to the number of mutations. In the breeds showing sequences too divergent, two consensus sequences were generated.

The analysis of consensus sequences per breed showed that for breeds such as Achai, Bhagnari, Red Sindhi, N'Dama, Brangus, and Dhanni, the sequences had at least two major lineages. The phylogenetic analysis confirmed this pattern (Figure 7), since they group in different branches. The same was seen for goat and sheep sequences. The phylogenetic analysis of these consensus sequences for the cattle breeds produced four clusters: one with low divergency, including mostly *B. indicus* Asian breeds, a more diverse cluster with breeds from Africa, Asia and the mixed breed from America, one cluster containing only the American breeds, and a cluster with Dhanni and the second sequence of N'Dama. The same pattern was seen in the network analysis (Figure 8), showing the same clustering of the haplotypes. In these analyses, bison and yak formed one cluster and water buffalo was a singleton. Goats and sheep formed two related clusters.

**Figure 9:**
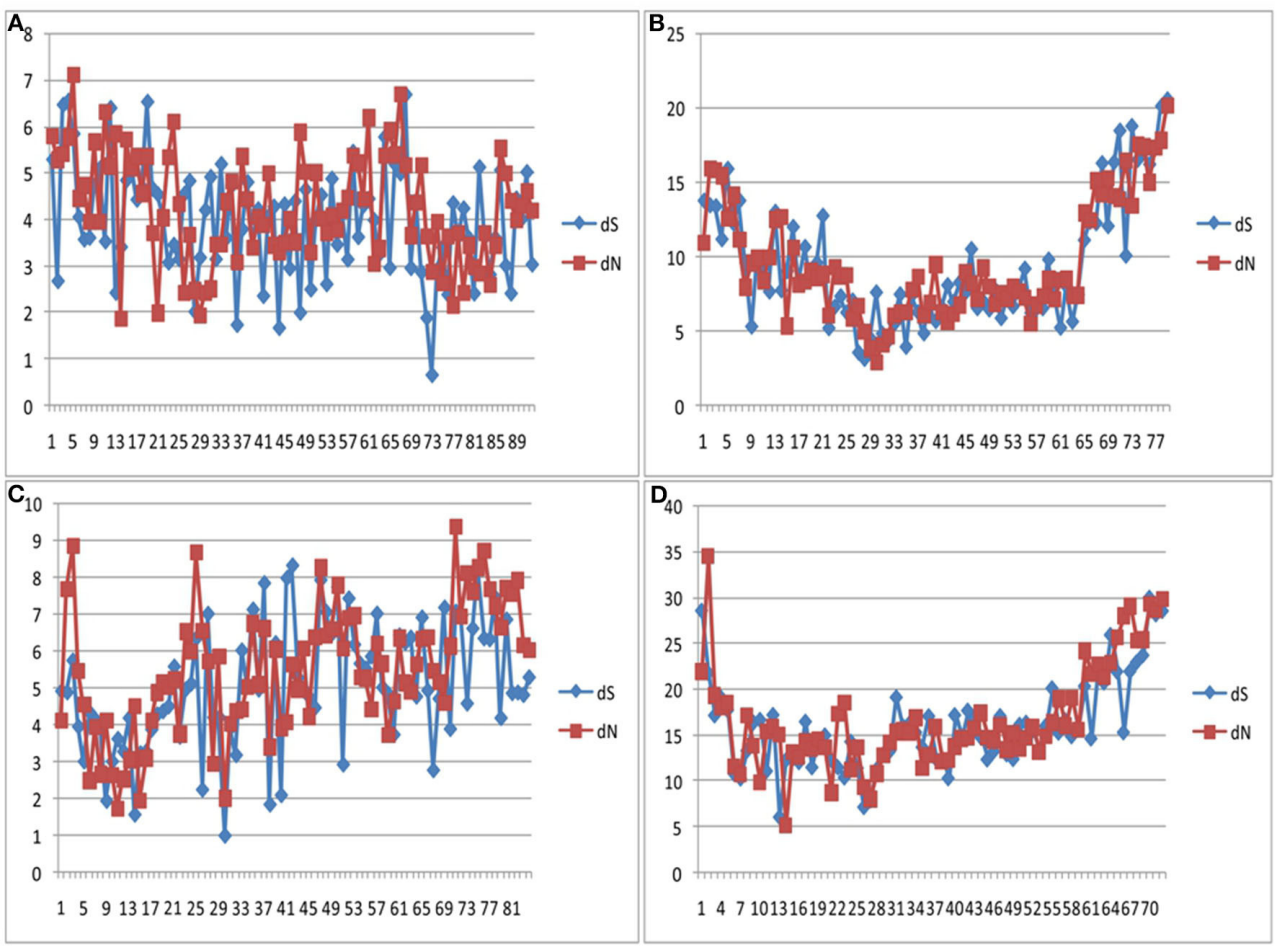
dS and dN values at the coding region of the bovine IFNAA gene showing positive selection for the African **(A)**, Asian **(B)**, American **(C)** breeds, as well as all breeds combined **(D)**.

Codon-based Z test, using the Nei-Gojobori method, revealed that the bovine IFNAA gene was under positive selection for variation (Table 4), since *Z*-values were highly significant (*p* > 2.58, *p* > 0.01). Even though variation was found to be non-homogeneous throughout the analyzed region (Figure 9), showing more variation at the beginning and the end of the region, the dN and dS values were very similar (ratio around 1) in the whole region.

**Table 4:** Codon-based *Z*-test parameters using the Nei-Gojobori method as described in the text.

**Continent**	**dS**	**dN**	**dN/dS**	**Z-statistic**
Africa	3.95 ± 0.12	4.26 ± 0.12	1.08	3.861
Asia	9.45 ± 0.47	9.63 ± 0.44	1.02	6.681
America	5.08 ± 0.18	5.48 ± 0.19	1.08	5.974
Combined	16.03 ± 0.59	16.75 ± 0.67	1.04	7.663

Forty-four nsSNPs of the IFNAA alleles were obtained from the alignment of the deduced amino acid sequences of cattle (Table 5). Eight and twelve were predicted to be deleterious by PROVEN and PolyPhen 2, respectively. The two algorithms identified T29C, N34L, N34T, and M39H as being harmful.

**Table 5:** Predicted effect of amino acid substitutions observed on the function of IFNAA protein.

**Substitutions**	**PROVEAN**		**PolyPhen-2**	
	**Prediction**	**Score**	**Prediction**	**Score**
T29S	Neutral	−1.129	Benign	0.000
T29L	Deleterious	−3.538	Benign	0.021
T29A	Deleterious	−2.611	Benign	0.000
T29C	Deleterious	−3.684	Possibly damaging	0.717
A33P	Neutral	−1.633	Benign	0.001
A33L	Neutral	−1.035	Benign	0.000
A33C	Neutral	−1.756	Possibly damaging	0.616
A33H	Neutral	−1.464	Benign	0.002
N34L	Deleterious	−5.141	Possibly damaging	0.930
N34T	Deleterious	−2.778	Possibly damaging	0.928
N34P	Deleterious	−4.326	Benign	0.399
N34H	Neutral	−2.377	Possibly damaging	0.561
M39T	Neutral	−0.070	Benign	0.000
M39P	Deleterious	−2.510	Benign	0.167
M39Q	Neutral	−1.624	Benign	0.027
M39H	Deleterious	−2.760	Possibly damaging	0.492
E60A	Neutral	1.535	Benign	0.001
E60P	Neutral	−0.778	Benign	0.190
E60L	Neutral	−1.780	Benign	0.008
E60S	Neutral	0.881	Benign	0.008
L62P	Neutral	8.779	Benign	0.001
L62C	Neutral	0.033	Probably damaging	0.963
L62A	Neutral	2.090	Benign	0.002
L62Q	Neutral	1.752	Possibly damaging	0.712
P97A	Neutral	1.759	Benign	0.003
P97L	Neutral	−0.852	Benign	0.013
P97S	Neutral	3.919	Benign	0.004
P97F	Neutral	−0.511	Benign	0.003
P97Q	Neutral	0.571	Benign	0.038
T99A	Neutral	3.389	Benign	0.000
T99L	Neutral	−1.127	Benign	0.016
T99S	Neutral	0.767	Benign	0.020
T99F	Neutral	−2.142	Possibly damaging	0.651
T99Q	Neutral	−1.022	Benign	0.211
K102E	Neutral	0.158	Benign	0.002
K102T	Neutral	0.277	Benign	0.010
K102Q	Neutral	0.057	Benign	0.007
K102H	Neutral	−1.492	Benign	0.036
R109G	Neutral	−1.213	Possibly damaging	0.531
R109W	Neutral	−0.179	Probably damaging	0.968
A110T	Neutral	2.810	Benign	0.000
A110D	Neutral	−0.633	Benign	0.001
A110G	Neutral	−1.552	Benign	0.012
A110Q	Neutral	−0.952	Possibly damaging	0.523

*A, alanine; K, lysine; E, glutamic acid; T, threonine; L, leucine; F, phenylalanine; Q, glutamine; S, serine; R, arginine; G, glycine; D, aspartic acid; N, asparagine; P, proline; W, tryptophan; H, histidine; M, methionine*.

## Discussion

This study represents the first genomic analysis of the type I IFNA genes in members of Bovidae family, showing some common evolutionary patterns of duplications/deletions at the lower taxa level, but also species-specific patterns. In this sense, the phylogenetic analysis of type I IFN genes from fishes all the way to mammals showed rapid evolutionary pattern through both substitutions and gene birth-death process since the origin of the tetrapods, as a way to carry out a host-pathogen arms race with viruses (31). One hypothetic evolutionary pathway of the IFNs propose one of the IFNA genes as the likely ancestor of the IFNBs by duplication in reptiles, that later evolved into IFNE and IFNK, early in the evolution of mammals, while some IFNAs evolved into the other subtypes (32). However, besides gene duplication, gene conversion has been proposed to be one important mechanism for type I IFN diversification. Several cases of gene conversion have been suggested in porcine IFNA, IFND and IFNW genes, including tentative regulatory sequences as well as the coding ORFs (33). Both gene conversion and duplications have also been reported in human IFNA genes, but the latter is suggested to have led to the creation of eutherian IFNA gene families, while gene conversion could have contributed to both creating and maintaining species-specific phylogenetic clustering (34). In our study, the presence of many examples of high identity between genes of the same species is a good indication of multiple events of gene duplication/conversion among the Bovidae species, although between cattle and bison more homologous relationships were established.

The large expansion of type I IFN genes in Bovidae is likely the result of a strategy to better adapt to higher diversity of viral challenges due to their environment. This have been suggested in pig, since there has been also an increase in the number of genes in its genome that could maximize their functional spectrum to confer subtype- and isoform-specific antiviral activity against different virus species (33). Although some functional overlaps of the type I IFNs are present, natural selection unlikely would select for such a wide variety of IFN proteins unless each one fulfilled unique functions or roles during different types of immune responses (35).

The phylogenetic relationships among the bovine type I IFN genes show a very similar evolutionary pattern to the one previously reported for this species (10), with the same clear distinction of the different subtypes. The number of some subtypes do not match, due to the use of different assemblies, which is expected since we used the latest version representing a much improved genome annotation of the genes, since it represents a 200-fold improvement in sequence continuity and a 10-fold improvement in per-base accuracy over previous cattle genome assemblies (16). The proposed IFNX subtype in the earlier study (10) is recoded in our study as IFNAL and IFND subtypes, which have high identity with genes of the other members of the Bovidae family, as well as in pigs.

Studies have shown that haplotype diversity can substantially reveal the amount of phenotypic variance at a particular genomic region (36). It is important to study the genetic basis of the mechanisms for the evolution and maintenance of genetic variation in the innate immune system among or within species, since the functions of the IFNA genes are critical for triggering the immune response to a viral infection or other immune pathogenic states. High level of polymorphism have been found in other genes in cattle, such as the genes of the major histocompatibility complex, which is proposed to be caused by diversifying selection, in order to increase adaptability under environments where the animals are challenge by different pathogens (17, 37, 38).

It has been repeatedly shown that loss of genetic variation can lead to short-term reduction of fitness components such as survival, reproductive output, and growth rates, and that genetic diversity plays an important role in buffering populations against pathogens and widespread epidemics (39), and the most widely used methods of assessing the genetic diversity are through the haplotype and nucleotide diversity indices. Our study provides a significant contribution in the characterization of the genetic variation of one of the IFNA genes. Here, the fact that haplotypes counts were significantly higher in 12 out 18 breeds in this study for the IFNAA gene, can be taken as a good indication of the existence of high haplotype diversity in the IFNA genes in general, both within and between breeds. This high level of genetic diversity may be attributed to evolutionary adaptation due to diverse environmental and disease exposures.

Among the African breeds of cattle, N'Dama and White Fulani showed the highest estimated diversity at the IFNAA locus, presenting high values of S and k. Our findings for N'Dama agreed with other studies showing highly divergence of this breed, compared to other African and European breeds (17, 40). This may be attributed to its disease tolerance and ability to endure harsh environment, which is thought to have developed over time due to evolutionary forces affecting the modulation of innate immune response of the animals (41). The high level of diversity of the White Fulani breed could be related to the type of farming, since the traditional management of this breed is done by migration through large geographical areas in search of adequate pasture and water due to the harsh environment they live in (42).

Even though the number of animals analyzed of the American breeds were small (except for Brangus), they all showed high average haplotype counts, compared to their African and Asian counterparts, and their sequences were highly divergent. This is consistent with a previous study on the major histocompatibility complex gene DRB3, where Hereford and Brangus showed high genetic variability and the former showed also high divergence compared to the other breeds studied (17). This could be due to the high selection pressure that the American breeds are subjected to.

As seen in this study, non-synonymous substitution was generally higher than the synonymous substitution meaning there are many amino acid changes that may influence differential gene expression or structural and functional changes in IFN proteins, thereby affecting its binding activities and other immunological consequences. Likewise, the high diversity of haplotypes can generate additional additive effects on the gene expression. For instance, many of these variants may lead to reduce disease resistance or increase susceptibility to bacterial and viral infections or vice versa. Yu et al. (43) reported that the expression of intracellular human IFNA2 conferred antiviral properties in transfected bovine fetal fibroblasts and did not significantly affect the full development of somatic cell nuclear transfer embryos.

The estimates of Tajima's *D*-value for all the breeds, except Brangus, Holstein, and Lohani, were negative, although only the values for Bhagnari and Cholistani were statistically significant, indicating the presence of excessive rare alleles and that there are more variation than would be expected from a population in Hardy-Weinberg equilibrium (22). This could mean that both natural and artificial selection is selecting for higher levels of genetic variation in the IFNAA locus among these populations. On the other hand, positive selection may be also acting in this gene from a higher dN/dS ratio found in the study for this region. In this sense, Peters et al. (17), found similar results, analyzing the same breeds but for the major histocompatibility complex class II gene DRB3.

A study of 15 autosomal genes in 14 representatives of the Bovinae subfamily, has reported higher proportions of dN/dS in cattle, proposing to be the result of the domestication process, although the frequency of polymorphism in this species, compared to the other species of the tribe Bovini which have very similar dN/dS ratio, suggesting that the evolutionary rate in these genes has remained stable, even though they are subject to positive selection (44). It can be inferred that the IFNAA gene in the cattle breeds studied is under a selection process since disease resistance improves productivity and, therefore, is selected positively. Purifying selection, rather than positive selection, has been reported in pigs using pairwise comparison of multi-gene subtypes, as well as by analyzing within gene variation, in IFNDs and IFNWs, whereas more positive selection pressure was detected among the genes of IFNA subtype (33). Positive selection could be the result of disease pressure from viral infection which could affect more IFNA genes, while purifying selection could be the result of fixation of immune or development regulation (6).

Our study revealed the first evidence of IFNAA haplotype-base framework in cattle, which could be used in association studies of different disease phenotypes. Several reports have shown that association studies with haplotype analysis are a powerful tool to elucidate genetic mechanism of variations underlying complex disease traits (36, 45–47).

According to the pairwise F_ST_ values for the breeds studied, there is a low genetic differentiation in the breeds within the same continent, so it is assumed that the IFNAA sequences are more conserved within the same region, which could be associated to their regional adaptation to the same environmental conditions and disease pathogens conditioning their immune systems. Similar results were found in a study with breeds of Pakistani cattle using microsatellites, as well as in a previous study of the same breeds analyzed in our study, but using the sequences of the DRB3 gene (17, 48), where low values of pairwise F_ST_ were reported in the breeds of cattle from the same region. These studies also found that the beef breeds Angus and Hereford are genetically closer to each other than to the dairy breeds, suggesting that breed selection has affected not only genes related to the type of production, but also the majority of the genes in the genome.

The dendrogram constructed in our study suggest that most breeds showed a separated evolutionary pattern, with some breeds having diverging for a long time. American breeds appeared to be closest to the origin in the evolutionary trend, along with Dhanni breed. The tree also suggests that Nari master, Lohani, Dajal, Cholistani, Tharparkar, and one haplotype group of Achai, Red Sindhi, and Bhagnari (Asian breeds) shared a more recent common ancestor with Sokoto Gudali (African breed) than to the other cattle breeds from Africa and America studied here. The relationship along each clade may uncover the underlying haplotypes variations related to ancestral disease variants and immune responses. Also, the breeds did not cluster strictly based on *Bos taurus* or *Bos indicus*, since yak, and bison showed haplotypes highly similar to certain breeds of cattle.

In this study, four amino acids changes could predict potential regions for putative disease associated variants. These amino acid substitutions with negative functional effects on IFNAA protein may be associated with variation in both innate and adaptive immune responses and different disease phenotypes among the breeds in this study. These may have pathological phenotypic consequences (49, 50), of which, the N34T seems to be more deleterious (−5.141 for PROVEAN and 0.930 for PolyPhen-2). With IFNAA gene being known to be involved in signal transduction and ligand binding, this kind of variation may induce structural and functional defects accompanied with significant immunomodulatory perturbation and consequences on the target cells.

Normal protein function can be changed by deleterious nsSNPs, through disruption of salt bridges or hydrogen bonds (51), hydrophobic changes (52), and geometric constraint changes (53). The differences in prediction capabilities of PROVEAN and PolyPhen-2 used in this study may be due to their differing alignment procedures. De Alencar and Lopes (54) reported that difference in the results of computational tools may be as a result of differences in features utilized by the tools and therefore dissimilar predictions might be expected.

## Conclusions

Our study shows a detailed evaluation of the type I interferons in the bovine genome, showing the evolutionary pattern among these genes, but also among the alpha subtype within the Bovidae family. We also have provided the first comprehensive genetic variation of the IFNAA locus in different breeds of cattle from three continents, thereby providing an insight into the global geographical distribution of the variation in this gene, which is very likely to have an influence in economically important traits. Genetic diversity reported in this study is more pronounce within breed than between breeds, while there is no well-differentiation considering diversity across continental breeds, nor species within the Bovinae subfamily. The results found for IFNAA could be a good indication of what is happening in all the alpha interferons for these species.

Based on the findings of this study, we suggest a further study to associate the diversity in IFNAA with disease phenotypes which could be harnessed for resistance/tolerance against bacterial and viral infection in cattle.

## Data Availability Statement

The datasets presented in this study can be found in online repositories. The names of the repository/repositories and accession number(s) can be found in the article/Supplementary Material.

## Ethics Statement

This animal study was reviewed and approved by Association for Assessment and Accreditation of Laboratory Animal Care International (AAALAC).

## Author Contributions

SOP, TH, and AA conceived the project, and designed and performed the experiments. SOP, TH, MD, OM, SP, JH, MB, AY, and II analyzed the data. SOP, TH, MD, and II wrote the manuscript. JH, MB, OM, and AY contributed to the scientific content. All authors read and approved the final manuscript.

## Conflict of Interest

The authors declare that the research was conducted in the absence of any commercial or financial relationships that could be construed as a potential conflict of interest.
