# Supplementary material for: Evolutionary Pattern of Interferon Alpha Genes in Bovidae and Genetic Diversity of IFNAA in the Bovine Genome
Source: Front Immunol. 2020 Sep 30;11:580412. doi: 10.3389/fimmu.2020.580412 (PMC7561390; doi:10.3389/fimmu.2020.580412)
Supplement: Supplementary file 1 [file Table_1.docx]

**Supplemental Table 1:** Recoded names of the bovine type I interferon genes according to their phylogenetic relationship, showing their Gene and protein IDs.

| **Recoded Name** | **Gene ID** | **Protein Accession** |
| --- | --- | --- |
| IFNAA | 515951 | NP_001017411 |
| IFNAB | 100335490 | XP_003586414 |
| IFNAC | 281236 | NP_776510 |
| IFNAD | 100329204 | XP_005209953 |
| IFNAE | 783912 | XP_002689578 |
| IFNAF | 100329210 | NP_001165513 |
| IFNAG | 100329206 | NP_001165511 |
| IFNAH | 100329207 | NP_001165512 |
| IFNAI | 510726 | XP_002689601 |
| IFNAJ | 618947 | XP_002689560 |
| IFNAK | 523244 | XP_010806062 |
| IFNAL1 | 787343 | XP_002689584 |
| IFNAL2 | 517108 | XP_010806071 |
| IFNB1A | 281845 | NP_776775 |
| IFNB1B | 112441471 | XP_024851835 |
| IFNB2A | 517016 | NP_001015617 |
| IFNB2B | 525550 | XP_002689587 |
| IFNB2C | 784525 | XP_010806071 |
| IFNB4 | 616977 | XP_024851834 |
| IFNB3A | 618946 | NP_001107769 |
| IFNB3B1 | 100848709 | XP_024852161 |
| IFNB3B2 | 112441480 | XP_024852165 |
| IFNB3B3 | 112447877 | XP_024852162 |
| IFNB3B4 | 112447878 | XP_024852163 |
| IFNB3B5 | 112447879 | XP_024852164 |
| IFNB3B6 | 112447880 | XP_024852166 |
| IFND | 104969295 | XP_024852159 |
| IFNE | 101904725 | XP_005209958 |
| IFNK | 100138192 | NP_001193352 |
| IFNT1 | 528513 | NP_001026935 |
| IFNT2 | 317698 | NP_001015511 |
| IFNT3 | 100313956 | NP_001161747 |
| IFNW1 | 281847 | NP_776776 |
| IFNW2 | 781948 | XP_010806064 |
| IFNW3 | 515953 | XP_024851833 |
| IFNW4 | 104968438 | XP_003586416 |
| IFNW5 | 618801 | XP_002689555 |
| IFNW6 | 618985 | XP_002689573 |
| IFNW7 | 617112 | XP_005209955 |
| IFNW8 | 618859 | XP_010806066 |
| IFNW9 | 617135 | XP_002689569 |
| IFNW10 | 781853 | XP_005209954 |
| IFNW11 | 781778 | XP_010806070 |
| IFNW12 | 100298573 | XP_010806067 |
| IFNW13 | 100298530 | XP_010806068 |
| IFNW14 | 618943 | XP_002689576 |
| IFNW15 | 509166 | NP_001232865 |
| IFNW16 | 100299481 | XP_002689578 |
| IFNW17 | 100847720 | XP_003586413 |
| IFNW18 | 619113 | XP_002689554 |
| IFNW19 | 513706 | XP_015327912 |
| IFNW20 | 515485 | XP_010806063 |
| IFNW21 | 101908039 | XP_015327910 |
| IFNW22 | 107132683 | XP_015327909 |
| IFNW23 | 523509 | XP_002689580 |
