# Supplementary material for: Evolutionary Pattern of Interferon Alpha Genes in Bovidae and Genetic Diversity of IFNAA in the Bovine Genome
Source: Front Immunol. 2020 Sep 30;11:580412. doi: 10.3389/fimmu.2020.580412 (PMC7561390; doi:10.3389/fimmu.2020.580412)
Supplement: Supplementary file 2 [file Table_2.docx]

**Supplemental Table 2:** Haplotypes distribution of the bovine IFNAA gene among the studied cattle breeds.

| **Breed** | **Origin** | **N** | **Haplotypes** | | | | | | | | | | | | | | | | | | | | | | **AHC** |
| --- | --- | --- | --- | --- | --- | --- | --- | --- | --- | --- | --- | --- | --- | --- | --- | --- | --- | --- | --- | --- | --- | --- | --- | --- | --- |
|  |  |  | **1** | **2** | **3** | **4** | **5** | **6** | **7** | **8** | **9** | **10** | **11** | **12** | **13** | **14** | **15** | **16** | **17** | **18** | **19** | **20** | **21** | **22** |  |
| Achai | Pakistan | 13 | 0 | 0 | 3 | 4 | 1 | 3 | 0 | 1 | 1 | 0 | 0 | 0 | 0 | 0 | 0 | 0 | 0 | 0 | 0 | 0 | 0 | 0 | 0.462 |
| Angus | USA | 3 | 2 | 1 | 0 | 0 | 0 | 0 | 0 | 0 | 0 | 0 | 0 | 0 | 0 | 0 | 0 | 0 | 0 | 0 | 0 | 0 | 0 | 0 | 0.667 |
| Bhagnari | Pakistan | 15 | 0 | 0 | 0 | 3 | 2 | 2 | 3 | 1 | 1 | 1 | 0 | 1 | 0 | 0 | 0 | 0 | 0 | 1 | 0 | 0 | 0 | 0 | 0.600 |
| Brangus | USA | 14 | 5 | 2 | 0 | 1 | 0 | 0 | 0 | 0 | 1 | 0 | 1 | 1 | 0 | 0 | 2 | 0 | 1 | 0 | 0 | 0 | 0 | 0 | 0.571 |
| Cholistani | Pakistan | 17 | 0 | 0 | 1 | 13 | 1 | 0 | 0 | 0 | 1 | 0 | 0 | 0 | 1 | 0 | 0 | 0 | 0 | 0 | 0 | 0 | 0 | 0 | 0.294 |
| Dajal | Pakistan | 17 | 0 | 1 | 1 | 11 | 0 | 0 | 0 | 0 | 1 | 0 | 0 | 0 | 1 | 0 | 0 | 0 | 0 | 0 | 1 | 0 | 1 | 0 | 0.412 |
| Dhanni | Pakistan | 15 | 0 | 0 | 0 | 0 | 0 | 0 | 0 | 0 | 0 | 0 | 0 | 0 | 0 | 0 | 1 | 0 | 1 | 1 | 2 | 1 | 4 | 5 | 0.467 |
| Hereford | USA | 3 | 2 | 0 | 0 | 1 | 0 | 0 | 0 | 0 | 0 | 0 | 0 | 0 | 0 | 0 | 0 | 0 | 0 | 0 | 0 | 0 | 0 | 0 | 0.667 |
| Holstein | USA | 6 | 0 | 1 | 0 | 0 | 0 | 0 | 0 | 0 | 0 | 0 | 0 | 1 | 0 | 1 | 3 | 0 | 0 | 0 | 0 | 0 | 0 | 0 | 0.667 |
| Lohani | Pakistan | 16 | 0 | 0 | 1 | 9 | 0 | 0 | 0 | 0 | 0 | 1 | 0 | 0 | 0 | 0 | 1 | 0 | 2 | 2 | 0 | 0 | 0 | 0 | 0.375 |
| Muturu | Nigeria | 8 | 0 | 0 | 0 | 4 | 0 | 0 | 0 | 0 | 1 | 1 | 0 | 1 | 0 | 0 | 1 | 0 | 0 | 0 | 0 | 0 | 0 | 0 | 0.625 |
| Nari Master | Pakistan | 8 | 0 | 0 | 2 | 5 | 0 | 0 | 0 | 0 | 0 | 1 | 0 | 0 | 0 | 0 | 0 | 0 | 0 | 0 | 0 | 0 | 0 | 0 | 0.375 |
| N’Dama | Nigeria | 15 | 0 | 1 | 0 | 3 | 0 | 0 | 0 | 0 | 0 | 0 | 0 | 1 | 0 | 0 | 0 | 0 | 0 | 2 | 0 | 7 | 1 | 0 | 0.400 |
| Red Sindhi | Pakistan | 13 | 0 | 0 | 0 | 2 | 2 | 2 | 2 | 1 | 0 | 0 | 0 | 1 | 0 | 0 | 0 | 3 | 0 | 0 | 0 | 0 | 0 | 0 | 0.538 |
| Sahiwal | Pakistan | 8 | 0 | 0 | 0 | 5 | 0 | 0 | 0 | 0 | 0 | 1 | 0 | 1 | 1 | 0 | 0 | 0 | 0 | 0 | 0 | 0 | 0 | 0 | 0.500 |
| Sokoto Gudali | Nigeria | 15 | 0 | 0 | 2 | 6 | 1 | 0 | 1 | 1 | 1 | 0 | 0 | 1 | 0 | 0 | 0 | 0 | 2 | 0 | 0 | 0 | 0 | 0 | 0.533 |
| Tharparker | Pakistan | 13 | 0 | 0 | 0 | 5 | 1 | 2 | 0 | 1 | 0 | 1 | 0 | 0 | 0 | 2 | 0 | 1 | 0 | 0 | 0 | 0 | 0 | 0 | 0.538 |
| White Fulani | Nigeria | 14 | 1 | 0 | 0 | 2 | 0 | 0 | 0 | 0 | 0 | 2 | 2 | 2 | 2 | 2 | 1 | 0 | 0 | 0 | 0 | 0 | 0 | 0 | 0.571 |
| Total number of individuals  per haplotype | | 213 | 10 | 6 | 10 | 74 | 8 | 9 | 6 | 5 | 7 | 8 | 3 | 10 | 5 | 5 | 9 | 4 | 6 | 6 | 3 | 8 | 6 | 5 | 0.103 |
| Total Number of breeds per haplotype | |  | 4 | 5 | 6 | 15 | 6 | 4 | 3 | 5 | 7 | 7 | 2 | 9 | 4 | 3 | 6 | 2 | 4 | 4 | 2 | 2 | 3 | 1 |  |

N: Number of sequences, AHC: Average haplotypes count
